# Prescriptions

**Published:** 1893-05

**Authors:** 


					﻿Prescription Department.
PAINFUL MENORRHAGIA.
The following is recommended in
menorrhagia with pain :
B. Tinct. hydrast. canad. .f § j.
Extr. hydrast. canad.. f 3 iv.
Sig.— Twenty drops three times a
day.—Il Raccoglitore Medico, u, 1890.
ENLARGED SCROFULOUS GLANDS.
B Iodoform..............gr.	xxx.
Balsam of Peru......3iss.
Glycerin..........	.. 3vj.
M. Sig.— For local use.—L' Union
Medicale.
CREASOTE IN TYPHOID.
A cheap, reliable, and effective meth-
od of prescribing creasote in typhoid
fever :
B Creasoti,
Glycerini...........aa3ij.
Alcoholis................ 3 iv.
M. Sig.—Four drops in water every
two hours.— Weekly Rev.
GASTRALGIA.
B Valerianate of Bismuth, | drachm.
Subnitrate of Bismuth. drachm.
Ext. Nucis Vomica. ....7 grains.
Ext. Gentian..............q.	s.
M. Sig.—Make fifty pills or capsules.
Two to four a day.
TONIC IN CONSUMPTION.
Dr. T. J. Mays suggests the follow-
ing as a stimulant to the appetite in
pulmonary consumption :
B Acid. Phosphoric Dilut.,
Acid. Muriatic Dilut.,
Acid. Sulphuric Dilut.,
Tinct. Ferri Chloridi.. aa f § ss
M. Sig.—Thirty drops in a half
glass of cold sweetened water during
meals.—Hospital Gazette, London, Eng-
land.
SELECT FORMULAE.
FOR REFLEX COUGH
For the treatment of the reflex cough
accompanying catarrhal sore throat,
there is no remedy so effective as a
spray of the following, which may also
be used as a gargle :
B Acidi Carbolici,..........3	j
Pulv. Sodii Bor. .......3	j
Cocaine Hydrochlor... . gr. xij
Glycerini Purif.........J	ss
Aquas Rosae........ad. Jxij
Sig.—To be used as directed.—
Ontario Med. Journal.
DIARRHOEA OF CHILDREN.
B Borax..................3 j.
Glycerini pur........3iv.
Tinct. corticis aurant.. mxlv.
Aq. destillat.......§ij.
M. Sig.—A teaspoonful every one
to three hours.—Med. World.
INTESTINAL DERANGEMENT.
Dr. Robert Coltmann, for indiges-
tion with flatulence and constipation,
often prescribes the following pill to
be taken just before meals :
B Pulv. podophyllin...............gr. j.
Acid carbolic............gr.	viij.
Gum asafoetida. .........gr.	xlviij.
Ext. taraxacum q. s. ft. pil. xvj—M
This combination he usually finds
successful in this class of cases, but
occasionally arsenic In Fowler’s solu-
tion gtt. ij before meals will be better.
ACUTE RHEUMATIC ATTACKS.
B Salicylic Acid..................3	iij
Sodium Bicarbonate........	3 ij
Elixir of Gaultheria............Ji
Spts. Vini. Gal................Ji
Antikamnia  ..................J ii
Water sufficient to make......J	iv
. Sig.—Dose, one fluid drachm every
hour or two.
				

